# Miscellany

**Published:** 1893-11

**Authors:** 


### Miscellany.

### CIVIL SERVICE EXAMINATIONS.

Open competitive examinations for the positions of junior assistant physicians and apothecaries in the State hospitals, will be held at the office of the Civil Service Commission, Albany, Thursday, November 16, 1893, at 10 o'clock A. M.

Applicants as junior assistant physicians must be residents of the State of New York, graduates of a legally incorporated medical college, and have had, at least, one year's actual experience on the staff of a public general hospital. Salary, $800 to $1,500 per annum and board.

Applicants as apothecaries must be residents of the State of New York, at least twenty-one years of age, and must have a license from the State Board of Pharmacy. Salary, $500 to $600 per annum and board.

For application blank, address New York Civil Service Commission, Albany, N. Y.

THOMAS CARMODY,

Albany, N. Y., October 20, 1893. *Chief Examiner.*

---

MESSRS. WILLIAM WOOD & Co., medical publishers of New York, announce the early publication of a System of Medical Jurisprudence and Toxicology, by R. A. Witthaus, A. M., M. D., Professor of Chemistry, Physics, and Hygiene in the University of the State of New York; and Tracy C. Becker, A. B., LL. B., Professor of Criminal Law and Medical Jurisprudence in the University of Buffalo.

This work will be sold by subscription only; muslin, $5.00 per volume; leather, $6.00. There are seventeen associated authors, three of whom are legal, and fourteen are medical. The plan of the system is such as to adapt it to the courts of the various states of America. Practically speaking, it is proposed that this work shall be so thorough as to supplant the requirement for any other text or guide-book for American practitioners of medicine or law. The system will consist of four octavo volumes, of about 600 pages each. These volumes will be printed in the best manner, from new type, and illustrated wherever desirable, by line and half-tone engravings and chromo-lithographic plates. In paper, press-work, and binding, it is promised that they will be specimens of the best work. The first volume may be expected to issue soon, and subscriptions should be placed at an early day. The prospects are that the first edition will be exhausted at an early day, as the prospectus is attracting the attention of doctors and lawyers in all English-speaking countries.

